# Control of the Alumina Microstructure to Reduce Gate Leaks in Diamond MOSFETs

**DOI:** 10.3390/nano8080584

**Published:** 2018-07-31

**Authors:** Marina Gutiérrez, Fernando Lloret, Toan T. Pham, Jesús Cañas, Daniel F. Reyes, David Eon, Julien Pernot, Daniel Araújo

**Affiliations:** 1 Faculty of Science, University of Cadiz, 11510 Puerto Real, Spain; fernando.lloret@uca.es (F.L.); jesus.canas@uca.es (J.C.); daniel.fernandez@uca.es (D.F.R.); daniel.araujo@uca.es (D.A.); 2 Institute for Material Research, University of Hasselt, 3590 Diepenbeek, Belgium; 3 UFR PHysique, Ingénierie, Terre, Environnement, Mécanique, Universite Grenoble Alpes, F-38042 Grenoble, France; thanhtoan.pham@fhnw.ch (T.T.P.); david.eon@neel.cnrs.fr (D.E.); julien.pernot@neel.cnrs.fr (J.P.); 4 CNRS, Institut NEEL, F-38042 Grenoble, France

**Keywords:** diamond, MOSFET, TEM, bandgap, dielectric functions, alumina, MPCVD

## Abstract

In contrast to Si technology, amorphous alumina cannot act as a barrier for a carrier at diamond MOSFET gates due to their comparable bandgap. Indeed, gate leaks are generally observed in diamond/alumina gates. A control of the alumina crystallinity and its lattice matching to diamond is here demonstrated to avoid such leaks. Transmission electron microscopy analysis shows that high temperature atomic layer deposition, followed by annealing, generates monocrystalline reconstruction of the gate layer with an optimum lattice orientation with respect to the underneath diamond lattice. Despite the generation of γ-alumina, such lattice control is shown to prohibit the carrier transfer at interfaces and across the oxide.

## 1. Introduction

Among the different wide bandgap semiconductors, diamond is considered to be intrinsically the best material for high-power devices due to its outstanding properties as the larger breakdown electric field (>10 MV/cm) [1] with the highest thermal conductivity (22 W/mm K) [2], high carrier mobility [3], high chemical inertness and thermal stability. However, due to the absence of large area substrates and to its relatively deep dopant levels (0.37 eV for boron and 0.57 eV for phosphorus) with respect to other semiconducting materials, industrial device applications have still not reached the market. Note that high-power applications mean high temperatures that can allow the improvement of the carrier concentrations and then the device performance. Indeed, working temperature around 200–300 °C is in the optimum temperature range for diamond [4].

Up to very recently, diamond field effect transistors (FETs) were usually based on a heavily boron-doped channel or a hydrogen-terminated (H-diamond) surface channel [5,6,7,8,9]. In both approximations, the carrier mobility is relatively low (<200 cm^2^/Vs), which motivates new geometries as bulk channel FET [10,11] where surface effects and impurity scattering (high doping) did not limit the device performance. In this more classical approach, the relatively high bandgap value of diamond makes it difficult to choose an adequate gate oxide if accumulation, as well as depletion mode, wants to be delivered. According to the bandgap values of different oxides, and their band settings with respect to diamond, alumina is a good candidate for diamond/oxide bandgap configuration [12,13]. However, the alumina bandgap value can lower down to 3.2 eV depending on its crystalline quality [14]. For alumina layers grown by atomic layer deposition (ALD) the bandgap seems to stand between 6 and 7.5 eV [15]. This value is close to that of the diamond bandgap, and thus, the crystallographic state of the gate oxide should be well controlled to prevent gate leaks or Fermi-level pinning due to oxide/diamond interface states.

**Figure 1:**
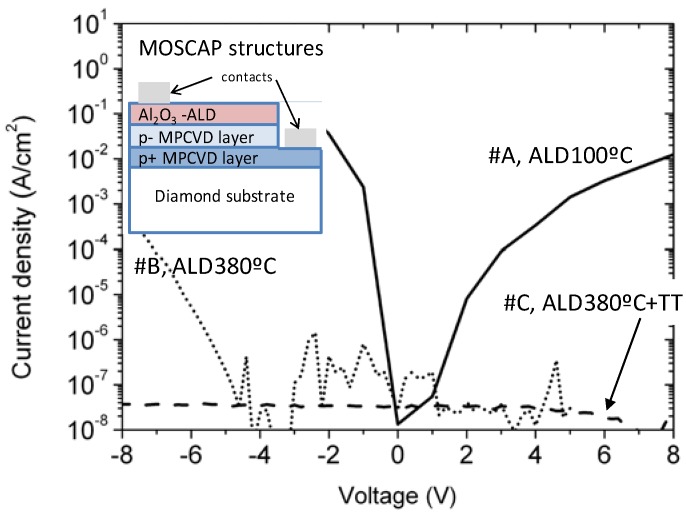
Representative I/V characteristics of the three samples under positive and negative bias. Sample A (ALD 100 °C) showed strong leakage current under positive and negative biases, while for sample B (ALD 380 °C), the leakage was strongly reduced, and sample C (ALD 380 °C + annealing at 500 °C for 30 min) showed no leakage current. MPCVD: microwave plasma-assisted chemical vapor deposition.

The alumina crystallography has been the object of countless studies for several decades as a result of its different stable and metastable phases. Aluminum oxide can have very different crystalline structures, degree of hydration, and defects, with the most technologically used being α-alumina, γ-alumina, and γ-Al(OH)_3_ aluminum hydroxide. The natural form of anhydrous alumina is called corundum (α-Al_2_O_3_), which is thermodynamically the most stable crystalline form, and its formation temperature ranges from 1050 to 1300 °C [16]. This has a rhombohedra structure where oxygen anions have almost hexagonal packing, and Al cations are in two-thirds of the octahedral interstitial positions. At the higher ALD deposition temperatures (400–500 °C), transition aluminas [17,18] (γ-aluminas) are the common states, which adapt their lattice parameters and symmetries to their compositions (i.e., they can be considered as a distorted spinel network (Mg^IV^Al^VI^_2_O_4_) with structural defects). The O anions take the place of a face centered cubic (FCC) lattice, while the Al cations occupy both the octahedral and tetrahedral coordination sites so that there are cationic vacancies that maintain the electrical neutrality of the set. Different concentrations of vacancies vary the stoichiometry, and 40–60% of Al and O, respectively, can be not conserved. Consequently, vacancies play an important role in the electrical properties of the oxide layers. The present contribution shows how the crystallography of the oxide gate has a strong influence on the electrical behavior of metal oxide semiconductor capacitor (MOSCAP) test structures. In particular, the growth temperature of the ALD and the posterior thermal annealing are shown to improve the gate-oxide crystallography that has good correspondence with the observed electrical behavior. A mechanism of the nanostructure modifications during annealing is proposed, which explains the observed electrical behavior of the MOSCAP (see Figure 1 inset) test structures.

## 2. Materials and Methods

The test structures are composed of a stack of a heavily (p+ layer) and a lightly (p− layer) boron-doped homoepitaxial mono-crystalline diamond layers grown by microwave plasma-assisted chemical vapor deposition (MPCVD) in a NIRIM type reactor with a 3 × 3 mm^2^ Ib high pressure and high temperature (HPHT) on (001) diamond substrates. The moderately boron-doped diamond layer (acceptor concentration, N_A_ ≈ 3 × 10^17^ cm^−3^) is in contact with the gate oxide. The underneath heavily boron-doped (N_A_ ≈ 5 × 10^20^ cm^−3^) metallic diamond p+ layer acts as a low resistive ohmic contact electrode in order to reduce the series resistance. Oxygen termination of the semiconducting diamond top layer was done thanks to a vacuum ultra violet (VUV) ozone treatment [11,19]. The Al_2_O_3_ gate oxide was deposited by ALD on the whole sample surface using a Savannah 100 deposition system from Cambridge NanoTech. The layer thickness was nominally 40 nm. The precursor was trimethylaluminium (TMA), and the oxidant was water. The pulse and the exposure time were 15 ms and 30 s, respectively, with the typical base pressure of 1.3 × 10^−1^ Torr. Two different ALD deposition temperatures were used, *T* = 100 °C for sample A and *T* = 380 °C for samples B and C. Sample C was further annealed at 500 °C for 30 min after the ALD deposition (Table 1). 

The ohmic and gate contacts were defined by laser lithography (Model: Heidelberg DWL66FS) and electron beam (e-beam) evaporation of Ti/Pt/Au (30/50/40 nm) followed by standard lift-off technique. The ohmic contact was deposited directly on the p+ layer. The studied devices are then the p+/p−/oxide/metal capacitor (MOSCAP) test structures that are used to evaluate the current–voltaje (I/V) behavior of the gate oxide layers.

## 3. Results

In Figure 1, the I/V behaviors of the three samples are compared. Ideally, the gate oxide should not have any leaks, but here as observed in a previously published work [20], electron tunneling into the oxide and hopping from trap to trap in the oxide, can reach the diamond through interface states. This mechanism explains the observed leaks in such MOSCAP structures. However, the presence of traps, fundamental to make possible such a leak mechanism, should still be evidenced. As it was observed in the continuous line, sample A showed some leaks in both biases, while sample B (i.e., higher ALD deposition temperature) leaked only for negative bias, and the leakage was much more reduced (dotted line). Sample C, which was the result of the thermal treatment (TT) (or annealing) of sample B (ALD + TT), showed that some leaks were below the detectivity of the setup (dashed line).

**Figure 2:**
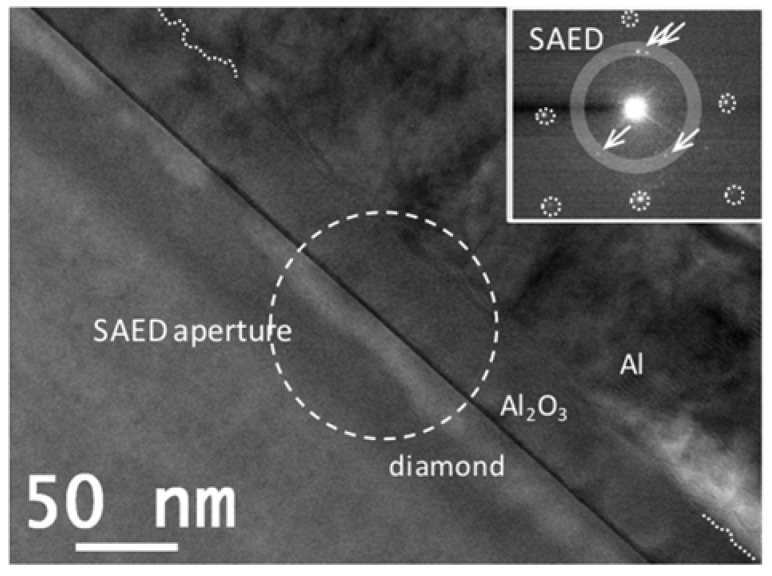
High-resolution electron microscopy (HREM) micrograph of sample A, where the followings is observed: The alumina layer is polycrystalline; Non-planar alumina top surface (dashed white line marks the top surface of the alumina in a specific area of the micrograph); and There is a thin layer of amorphous alumina between the diamond and the polycrystalline alumina area (identified by the white arrow).

According to the literature, microstructural studies [9,16,17,18], as well as chemical analysis, are required to understand the carrier transport through the interfaces and oxide (i.e., leaks). Concerning the interfaces, the bandgap value varies with the atomic configuration [14,15], which modifies the bandgap setting and then the carrier transport through the interface. Concerning the oxide itself, the grain boundaries promote carrier transport. For both aspects, the crystallography should be analyzed. Figure 2 shows the general observations by high-resolution electron microscopy (HREM) of sample A. At 100 °C ALD deposition, the layer was not smooth as indicated by the dashed line at the top of the oxide layer. The inset of this figure shows the selective area electron diffraction pattern (SAED) when the selective area aperture was located where the white dashed circle has been drawn. The spots furthest from the center (dashed circles in the SAED) correspond to the 100 zone axis diffractions of the diamond, while the rest of the spots come from diffractions of several alumina crystals. At least four spots inside the grey circles can be identified for the oxide layer corresponding to four grains inside the dashed circle of the aperture. Therefore, the clear polycrystalline character of the alumina layer is here well evidenced. In HREM observations (not presented in this contribution), the black contrast next to the diamond–alumina interface is identified as amorphous Al_2_O_3_ by the fast Fourier transform (FFT).

**Figure 3:**
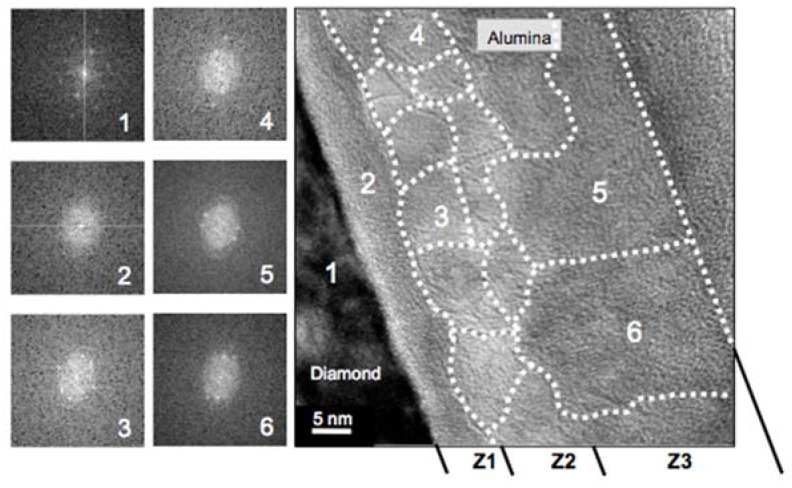
Low magnification HREM micrograph of sample B. On the left side the Fast Fourier transforms (FFTs) from six areas (labeled from 1 to 6) of the micrograph on the right are showed as an example of all those obtained on the whole micrograph. Three zones, labeled Z1, Z2, and Z3, are identified. Z1 corresponds to amorphous alumina (FFT 2), while Z2 and Z3 correspond to polycrystalline cubic alumina (FFT 3, 4, 5 and 6) where dashed white lines are used to delimit grains with different orientations.

In contrast, for the ALD deposition at 380 °C (sample B), the HREM studies identified three zones, labeled as Z1, Z2, and Z3, in the alumina layer (see Figure 3). Figure 3 is a high-magnification micrograph of an area of sample B where FFTs (left hand side insets) were carried out at the location indicated by a white number on the micrograph. Two different alumina crystallinity behaviors close to the interface with diamond were observed; the FFT of the alumina next to the diamond (Z1) showed an amorphous character, while in the rest of the alumina (zones Z3 and Z4), the related FFT patterns indicated a crystalline character. The grain boundaries (dashed white lines) were drawn as the result of applying the FFT all along the micrograph. This allowed us to define a region where the FFT pattern remained identical (i.e., one grain) and further to define the dashed lines corresponding to grain boundaries (i.e., grains with different orientation). Note that these observed grains defined here low angle grain boundaries, as several spots were repeated from FFT pattern to FFT pattern (see the left-hand insets of Figure 3). It is noteworthy that all the FFTs from Z2 and Z3 corresponded to an FCC crystal (i.e., the alumina grown by ALD at 380 °C was not the thermodynamically stable α-phase but the γ-phase).

Another remarkable aspect is the alumina grain size that varies from 5 to more than 20 nm. The carrier-hopping distance predicted by electrical measurements [20] was in this range, which allowed us to link this behavior to carrier hopping through the related states of the grain boundaries.

From such FFT analysis, the Z1 corresponded to a 5–8-nm-thick amorphous area while the Z2 and Z3 corresponded to polycrystalline areas. But, while in Z2, grain sizes below 15 nm were evidenced, in Z3, the grains were much larger. The FFT analysis of more than a hundred HREM micrographs provided another feature of this alumina layer; this is, in some locations the amorphous alumina layer was not observed, and the grains were directly in contact with the diamond. In those locations, the FFT pattern recorded on the alumina grain neighbor to the diamond corresponded to a 211-zone axis.

**Figure 4:**
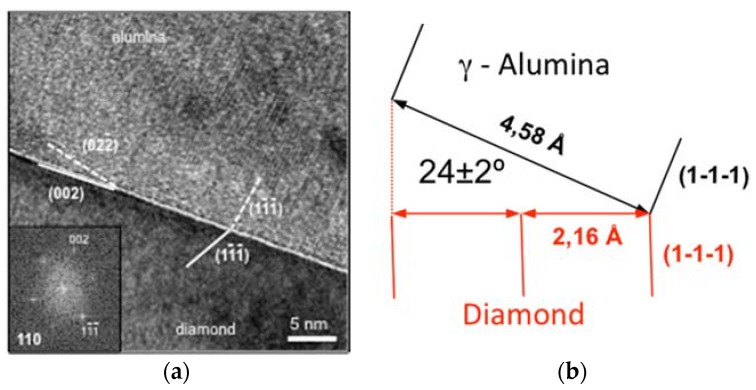
(**a**) HREM micrograph of sample C, where a fully crystalline alumina layer is observed. Lines and dashed lines were used to indicate where the $\left(1\bar{1}\bar{1}\right)$ planes are in both diamond and alumina crystals. The insets correspond to the FFTs of both materials; (**b**) Schematic representation of the $\left(1\bar{1}\bar{1}\right)$ planes’ correspondence at the diamond/alumina interface in sample C.

In sample C, the alumina layer configuration was shown to be fully monocrystalline, and the surface (not shown here) was atomically flat. The layer thickness was 40 nm as for sample A and B. The HREM micrograph in Figure 4a evidences the monocrystalline character of the oxide layer. The insets of Figure 4a correspond to the FFTs recorded at both the diamond and the alumina crystals regions. In the case of the diamond, as expected, the FFT corresponded to the 110-zone axis, while for the alumina, the 211-zone axis was identified. Both mentioned zone axes contained the $\left(1\bar{1}\bar{1}\right)$ plane reflection. Thus $\left(1\bar{1}\bar{1}\right)$ planes in diamond and alumina are showed up in the HREM observations. As it can be observed in the micrograph, $\left(1\bar{1}\bar{1}\right)$ planes in the diamond crystal (see continuous white line) rotated 24 ± 2° at the interface to become the $\left(1\bar{1}\bar{1}\right)$ plane in the alumina layer (white dashed line). This highlighted the strong lattice coherence between the diamond material and the oxide lattice.

In other samples with a layer thickness below 20 nm, the observed microstructure was amorphous. This shows that the formation of the nano-crystalline alumina grains needed a critical thickness around 20 nm to generate homogeneous crystallization during the ALD process. This is a key aspect if a further monocrystalline layer is needed to obtain after the thermal annealing.

## 4. Discussion

The rotation angle revealed in Figure 4 allowed the “junction” of both respective $\left(1\bar{1}\bar{1}\right)$ planes. Indeed, as the lattice interplane distances were 2.06 Å and 4.58 Å for the diamond and the γ-alumina, respectively, as it is shown in the schema of Figure 4b, this plane rotation allowed the fitting of both lattice. Unlike before the annealing (sample B), where amorphous alumina was observed, here, such lattice correspondence ensured a low diamond dangling bonds density and thus a low interface state levels density. Thus, the annealing here improved the crystallinity, from a polycrystalline to a monocrystalline character, and the interface state with the diamond material.

**Figure 5:**
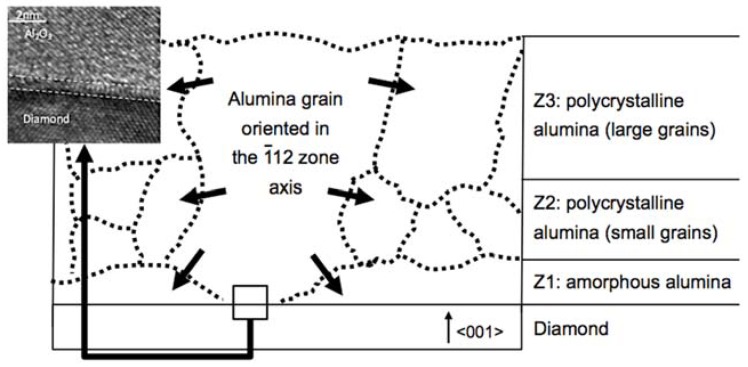
Schema of the grain evolution on γ-alumina grown on a diamond by ALD. The inset is a HREM micrograph of the alumina–diamond interface when amorphous alumina was not observed.

The crystallinity behavior during the annealing at 500 °C is tentatively explained by an up-to-down grain encroachment to diminish the system energy. Using electrical measurements on thin alumina layers, <20 nm, the MOSCAP structures always have leaks, even for high temperatures of ALD deposition and after annealing. As commented above, those observed in TEM always had an amorphous character, and thus, TEM and electrical behavior were consistent. When this thickness was above 30 nm, high ALD temperature growth and annealing improved the electrical behavior, as shown in Figure 1. In addition, the grain orientation observed above 30 nm was that of the further layer when annealing was performed. The authors propose the following mechanisms during the thermal annealing (Figure 5): (i) In some locations, as observed by TEM, a direct physical contact diamond/γ-alumina occurred at the interface, without the amorphous layer, with a grain orientation along the 211-zone axis; (ii) growing the layer sufficiently thick, this grain orientation persisted up to the top of the alumina layer and most of the top part of the layer had this orientation; and (iii) with annealing, the top part (Z3) with the larger grains (with respect to those of Z2) propagated down towards the diamond/alumina interface, encroaching on all the polycrystalline regions of the alumina layer. This resulted in the transformation of a polycrystalline alumina layer to a monocrystalline one.

Scanning transmission electron microscopy-energy dispersive spectroscopy (STEM-EDS) analysis was carried out before (sample B) and after the thermal annealing (sample C) to evaluate if the morphological lattice modifications induce variations in the stoichiometry of the alumina layer. No significant changes are observed before and after the annealing, as it was carried out in a vacuum atmosphere. The layers in both samples had a 60/40% O/Al atomic concentration ratio. A slight O content decrease of 5% from the surface towards the diamond/alumina interface is evidenced. Such a slight stoichiometry variation is possible as the phase is γ-alumina, i.e., a transition alumina. Thus, the oxygen atomic fraction increases close to the desired 60%, while the aluminum one drops to about 40% thanks to variations of vacancies. Such oxygen enrichment close to the surface occurs through oxygen diffusion from the surface, when after the ALD process was the sample is exposed to the air.

## 5. Conclusions

The control of the crystallinity was demonstrated to favor the electrical characteristics of the MOSCAP test structures. This can be obtained through posterior thermal annealing at 500 °C for 30 min. A relationship between the lattice configuration of the aluminum oxide and the diamond was shown to lower the interface states and gate leaks. The {111} planes of the diamond were shown to link up with those of the oxide layer with an angle of 24° ± 2°, which allowed the fitting of both lattice parameters. In addition, the annealing was also shown to form a monocrystalline oxide layer. Thus, this interface lattice fitting, which minimized the dangling bonds and the monocrystalline character after annealing, explained the improvement of the electrical behavior of the MOSCAP structure. The grain size observed before the annealing (sample B) corresponded to the carrier-hopping distance predicted in the literature [20], which links both aspects. In the latter, tunneling from trap to trap in the alumina layer was proposed as the main carrier transport mechanism for the leaks through the oxide layer. The distance from trap to trap estimated by the author was around 5 nm, which roughly corresponded to the observed grain size here. We concluded that those traps corresponded to dislocations located at the low angle grain boundaries observed before thermal annealing (sample B). The elimination of such crystalline defects and the improvement of the lattice fitting at the diamond/alumina interface reduced drastically the leaks.

## Figures and Tables

**Table 1:** Atomic layer deposition (ALD) and annealing temperatures for the studied samples.

Sample	ALD Temperature (°C)	Annealing Temperature (°C)/Time (min)
#A	100	Not annealed
#B	380	Not annealed
#C	380	500/30
